# Forecasting Obsolescence of Components by Using a Clustering-Based Hybrid Machine-Learning Algorithm

**DOI:** 10.3390/s22093244

**Published:** 2022-04-23

**Authors:** Kyoung-Sook Moon, Hee Won Lee, Hee Jean Kim, Hongjoong Kim, Jeehoon Kang, Won Chul Paik

**Affiliations:** 1 Department of Mathematical Finance, Gachon University, 1342 Seongnamdaero, Sujeong-gu, Seongnam-si 13120, Gyeonggi-do, Korea; ksmoon@gachon.ac.kr (K.-S.M.); lhw4540@gachon.ac.kr (H.W.L.); 2 Leo Innovision Ltd., #1906, IT Mirae Tower 33, Digital-ro 9-gil Geumcheon-gu, Seoul 08511, Korea; heejean.kim@dmsms.co.kr (H.J.K.); hoon@dmsms.co.kr (J.K.); cp@dmsms.co.kr (W.C.P.); 3 Department of Mathematics, Korea University, 145 Anam-ro, Seongbuk-gu, Seoul 02841, Korea

**Keywords:** components obsolescence, machine learning, forecasting, unsupervised clustering, diminishing manufacturing sources and material shortages

## Abstract

Product obsolescence occurs in every production line in the industry as better-performance or cost-effective products become available. A proactive strategy for obsolescence allows firms to prepare for such events and reduces the manufacturing loss, which eventually leads to positive customer satisfaction. We propose a machine learning-based algorithm to forecast the obsolescence date of electronic diodes, which has a limitation on the amount of data available. The proposed algorithm overcomes these limitations in two ways. First, an unsupervised clustering algorithm is applied to group the data based on their similarity and build independent machine-learning models specialized for each group. Second, a hybrid method including several reliable techniques is constructed to improve the prediction accuracy and overcome the limitation of the lack of data. It is empirically confirmed that the prediction accuracy of the obsolescence date for the electrical component data is improved through the proposed clustering-based hybrid method.

## 1. Introduction

A rapidly changing technological industry has caused the market to rapidly incorporate new materials and parts. This has caused product obsolescence to occur in every production line in the industry owing to the availability of products that achieve better performance or are more cost-effective or both. Strategies for addressing obsolescence are related to the expenses of firms and customer satisfaction. For the obsolescence management, reactive strategies such as lifetime buy, last-time buy, or identification of alternative parts are only temporary and may cause additional delays compared to the proactive strategies. If the probability of obsolescence and the cost associated with the obsolescence are high, it is recommended that one apply proactive management strategies to minimize the risk of obsolescence and associated costs. In fact, forecasting the occurrence of obsolescence is the key factor in proactive management, and many researchers have focused on the development of methods based on the prediction of obsolescence. Proactive strategies allow firms to prepare for the event of obsolescence; manufacturing losses can be reduced by predicting the life cycle of various components, including electronic components [1,2,3].

In this study, we aim to predict the cycle of diminishing manufacturing sources and materials shortages (DMSMS) obsolescence, which is defined as the loss of the ability to procure a technology or part from its original manufacturer. It is necessary to accurately predict the obsolescence cycle to reduce the risk for manufacturers and various companies caused by problems such as fast technology processes and short technology life cycles. Various statistical models for the accurate prediction of the obsolescence risk and date have been studied [4,5,6,7]. A Weibull-based conditional probability method as a risk-based approach to predicting microelectronic component obsolescence is described in [6]. The references to the problem of component obsolescence are summarized in [8]. However, it is difficult to implement a rapidly adapting statistical model to predict the obsolescence cycle of thousands of different types of components. Moreover, it is difficult to gather the input parameters of different models.

With recent improvements in computer performance, many methods for predicting future trends by learning large-capacity data and collecting necessary information are being studied. These learning methods, particularly machine-learning or deep-learning methods, are demonstrating outstanding results in various fields [9,10,11,12]. Depending on the data type or application, various machine-learning methods can be used. To the best of the authors’ knowledge, there are few studies in which these machine-learning or deep-learning methods have been applied to predict the cycle of DMSMS obsolescence. Jennings et al. (2016) [13] proposed two machine learning-based methods for predicting the obsolescence risk and life cycle. Good prediction results were reported by using random forest, artificial neural networks, and support vector machines for cell phone market data. Grichi et al. (2017, 2018) [14,15] proposed the use of a random forest and a random forest together with genetic algorithm searches for optimal parameter and feature selection for cell phone data, respectively. Trabelsi et al. (2021) [16] combined a feature selection and machine learning for obsolescence prediction. As described above, ordinary learning methods attempted to increase the accuracy of prediction by combining the existing machine-learning methods and applying them to the component obsolescence data. Although it is necessary to present efficient methods and hybridize them, it is expected that the accuracy of prediction can be improved further if the characteristics of each part data are used for learning. Therefore, in this study, the clustering method, which first classifies and learns data according to characteristics, is newly applied to predict the obsolescence of components.

The objective of this paper is as follows: Does machine learning improve the proactive strategy and prediction of obsolescence? Can it be effective and reliable? The obsolescence of the parts of diodes is predicted in this study when a sufficient amount of the data is not provided; the lack of available data for obsolescence problems is a crucial weakness in ordinary machine- or deep-learning methods. We propose a very accurate, fast, and reliable machine-learning method, which overcomes this weakness by using an unsupervised clustering algorithm and an ensemble of supervised regression techniques. Supervised regression tries to identify the parameters of the model from the labelled data and unsupervised clustering partitions the entire data into a few groups of similar data based on outward appearance. It is expected that the parameters obtained from a cluster of similar data fit machine-learning models better than the parameters from the entire set because the entire set has more variation and randomness. Thus, instead of constructing a single model for the entire set, several models are constructed, each of which is independently trained with the data in one cluster only, and the conjecture is experimentally validated by using several real datasets. It is the novelty of the study to apply an unsupervised clustering algorithm to supervised regression to improve model training. The usage of a hybrid ensemble method including several reliable regression techniques additionally improves the prediction accuracy; this is another novelty of the study. It is confirmed by using various measures that the prediction accuracy of the obsolescence date is improved through the proposed clustering-based hybrid method for diode data from three categories such as Zener diodes, varactors, and bridge rectifier diodes. The proposed clustering-based hybrid method can be easily extended not only to electrical component data but also to other types of obsolescence cycle prediction problems.

The rest of the paper is organized as follows. Section 2 describes the machine-learning and deep-learning algorithms used in the experiments. The proposed hybrid method based on *k*-means clustering is explained in Section 3. The statistics of the data and the descriptions of the hyperparameters are presented in Section 4. The accuracy measures and experimental results are presented and discussed in Section 5. The conclusions are drawn in Section 6.

## 2. Learning Models

It is important to choose a machine-learning or deep-learning algorithm with good predictive and computational performance for the dataset. For example, decision tree (DT) is a tree-building algorithm, which is easy to interpret and can adapt to learn complex relationships. An ensemble method can be constructed by combining several techniques into one that has better generalization performance than each individual algorithm. The two popular ensemble methods are bagging and boosting. We propose a hybrid method in this study and the merits of the proposed method are compared with those of various standard algorithms from individual algorithms (DT) to bagging algorithms (random forest), boosting algorithms (gradient boosting), and deep learning methods (deep neural network and recurrent neural network). We briefly introduce the following machine-learning and deep-learning algorithms and consider their combinations for improved results.

- Decision tree, random forest, gradient boosting
- Deep neural network, recurrent neural network

### 2.1. Decision Tree

The decision tree (DT) is a machine-learning method that is easy to understand and interpret and easy to use for both classification and regression. DTs based on features in training data start at the root of the tree and split the data based on the information gain. The following is used as the objective function to maximize this information gain in each division:

(1) $$f\left(parent,feature\right)=I_{p}-\sum_{j=1}^{n}\frac{N_{j}}{N_{p}}I_{j}.$$

Here, I is the impurity indicator, *N* is the number of samples of the node, the subscript ${}_{p}$ denotes the parent node, the subscript ${}_{j}$ denotes the *j*-th child node, and *n* is the number of child nodes. As an impurity indicator, entropy $I^{E}$ or Gini impurity $I^{G}$ is widely used,

$$I_{t}^{E}=-\sum_{i=1}^{m}\frac{N_{i}}{N_{t}}log_{2}\left(\frac{N_{i}}{N_{t}}\right),\;\;\;\;\;I_{t}^{G}=1-\sum_{i=1}^{m}{\left(\frac{N_{i}}{N_{t}}\right)}^{2}$$

where *m* is the number of classes in the node *t* and the subscript ${}_{i}$ denotes the *i*-th class in node *t*. DTs have a few restrictions on the training data; thus they are prone to overfitting. Therefore the maximum depth of the DT is usually controlled as a regulatory variable [10,11].

### 2.2. Random Forest

The random forest (RF) uses multiple DTs to improve prediction performance and reduces the risk of overfitting. First, a DT is trained by randomly selected samples from training data based on an objective function such as that in Equation (1). This process is then repeated several times to collect the prediction of each tree and make a decision by the majority vote method. When an RF splits the nodes of a tree, it finds the optimal features by considering randomly selected feature candidates among all features. This makes the tree more diverse and lowers the variance. Additionally, it is easy to measure the relative importance of a feature by checking how much a node using a certain feature reduces the impurity. The number of trees generated by an RF is a hyperparameter, and the larger the number of trees, the higher the computational cost, but the better the performance. Although an RF is more complex than DTs, it is more stable and can handle high dimensionality and multicolinearity better, being both fast and insensitive to overfitting [17,18,19].

### 2.3. Gradient Boosting

The gradient boosting (GB) method is used to train a set of predictors while complementing the previous model by sequentially adding classifiers. Starting from the leaf node of the DT, the estimate of the target is found from the argument that minimizes the sum of the loss functions. In other words, from the dataset ${\left\{\left(x_{i},y_{i}\right)\right\}}_{i=1}^{n}$, the prediction is first computed by

$$f_{0}\left(x\right)=argmin_{\gamma }\sum_{i=1}^{n}L\left(y_{i},\gamma \right).$$

For instance, with a differentiable loss function $L\left(y_{i},\gamma \right)={\left(y_{i}-\gamma \right)}^{2}/2$, we obtain the sample average $f_{0}\left(x\right)=\frac{1}{n}\sum_{i=1}^{n}y_{i}.$ The prediction is then sequentially updated by reducing the average of the pseudo residual as follows:

$$f_{m}\left(x\right)=f_{m-1}\left(x\right)+\nu \sum_{j=1}^{J_{m}}R_{jm}\left(x\right),$$

where $r_{im}=-\left(\frac{\partial L(y_{i},f_{m-1}\left(x_{i}\right))}{\partial f_{m-1}\left(x_{i}\right)}\right)$ is the residual of the data, $R_{jm}\left(x\right)$ is the average of the residuals $r_{im}$ that a sample *x* can be found in the *j*-th leaf node in the *m*-th tree, and $J_{m}$ is the number of leaf nodes of the *m*-th tree. Here, ν is the learning rate and is between 0 and 1, which reduces the effect of each tree and eventually improves the accuracy [11,20].

### 2.4. Deep Neural Network

**Figure 1:**
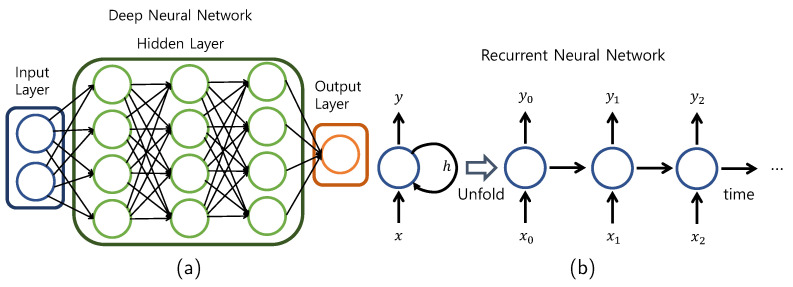
Structure of (**a**) a deep neural network and (**b**) a recurrent neural network.

Deep learning is based on artificial neural networks created by mimicking the principles and structure of human neural networks. In the human brain, neurons receive a certain signal, stimulus, etc., and when this stimulus exceeds a certain threshold, it is conceived in the process of transmitting the resulting signal. Here, the input stimulus and signal are input data from the artificial neural network, the threshold value is a weight, and the type of action performed by the stimulus can be compared to the output data. Hidden layers exist between the input and output layers, and the hidden layer uses an activation function to determine the optimal weight and bias. A learning method with two or more hidden layers is referred to as a deep neural network (DNN), as shown in Figure 1a. The computer creates a classification label on its own, distorts the space, and repeats the process of classifying data to derive the optimal dividing line [11,21].

### 2.5. Recurrent Neural Network

The recurrent neural network (RNN) algorithm is a type of artificial neural network specialized in repetitive and sequential data learning and contains an internal cyclic structure as shown in shown Figure 1b. By using a circular structure, past learning is reflected in the current learning through weights. It is an algorithm that solves the limitations of existing continuous, iterative, and sequential data-learning algorithms. It enables the connection of the present learning with the past learning and is time dependent [11,22].

### 2.6. Grid Search

**Figure 2:**
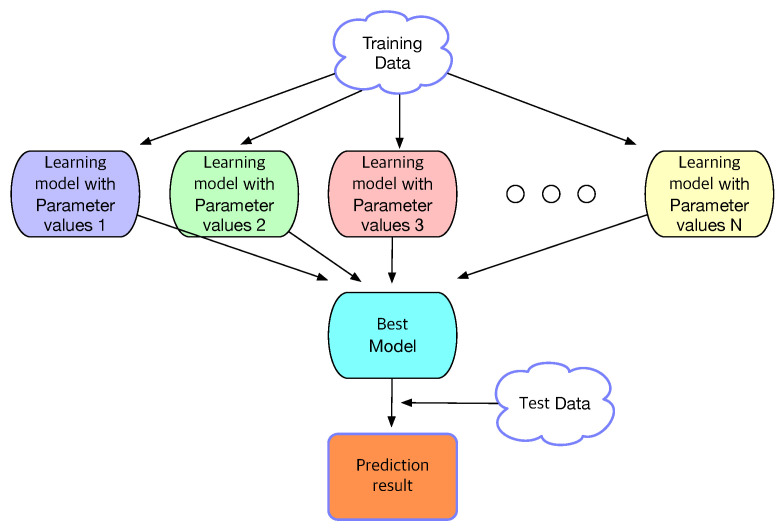
Flowchart of the grid search, which finds the right hyperparameters of a machine-learning model to achieve optimal performance.

For each machine-learning algorithm mentioned above, hyperparameter optimization is performed by using a grid search as shown in Figure 2 to determine the optimal parameters through which the best learning model is derived.

## 3. Hybrid Method

The machine-learning and deep-learning methods introduced in Section 2 can be applied as they are, but the prediction results can be further improved by grouping data with common properties. The *k*-means method from unsupervised learning is first introduced as a grouping method.

### 3.1. k-Means Clustering

A partition of a set $\left\{X_{1},X_{2},\cdots ,X_{n}\right\}$ in mathematics is a grouping of its elements into non-empty subsets $\left\{A_{1},A_{2},\cdots ,A_{k}\right\}$ in such a way that every element is included in exactly one subset. The *k*-means clustering is a method that aims to partition the observations into *k* clusters to minimize the variance of each cluster and distance difference. It is one of the unsupervised learning methods, which represent algorithms that learn patterns from unlabelled data.

**Figure 3:**
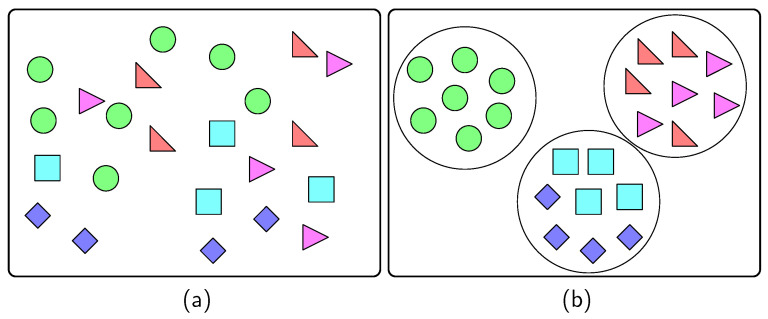
Data (**a**) before the partition and (**b**) after the partition with *k*-means clustering.

The detailed process is as follows. First, we randomly select *k* data and set this as the centroids of each cluster. All data are grouped to minimize the distance to *k* centroids. The centroid of the configured cluster is recalculated, and the above calculation is repeated until the cluster of each data does not change. That is, it is used to find *k* clusters that minimize the following variance,

$$\min_{D_{i}}\sum_{i=1}^{k}\sum_{x_{j}\in D_{i}}{\left||x_{j}-C_{i}|\right|}^{2}$$

where $C_{i}$ is the centroid of the *i*-th cluster $D_{i}$ with $D_{i}\cap D_{j}=\emptyset$ for i≠j. The proposed method performs the *k*-means method on the training data so that the unsorted data as in Figure 3a can be clustered into groups with certain similarities as in Figure 3b.

Although the *k*-means method has the advantage of improving the learning results, it also has limitations. First, the number of clusters *k* should be specified in advance, and depending on this value, different clustering results may be obtained. Additionally, there is a possibility that the error convergence of the algorithm converges to a local minimum rather than a global minimum, and it is sensitive to data outliers [10,23]. Because the number *k* of the clusters is a parameter dependent on the dataset, it is obtained by performing preliminary preprocessing as presented in Section 4.2.

**Figure 4:**
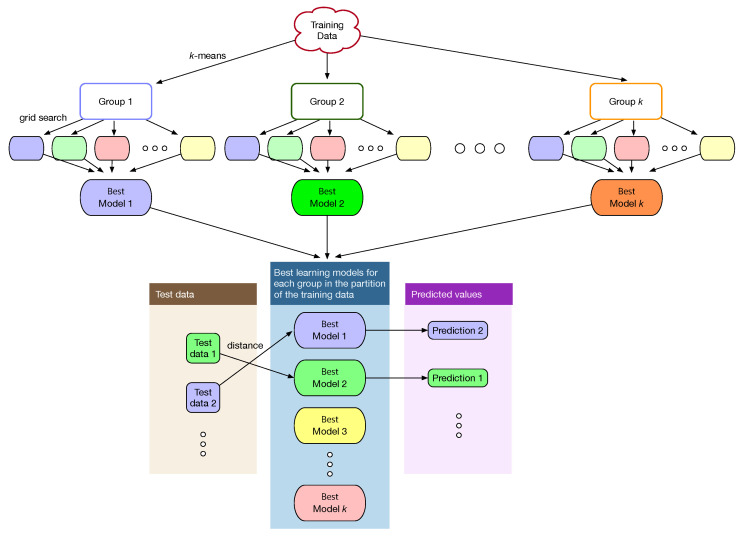
Determining the optimal hyperparameters of a machine-learning method for each cluster obtained by using unsupervised *k*-means clustering.

When machine learning is used for problem solving, a single model is constructed from the entire dataset. The *k*-means clustering algorithm in this study partitions the training data into disjoint clusters of similar data and multiple machine-learning models are then constructed, i.e., one model for each cluster. The grid search in Figure 2 is performed on each cluster as in Figure 4 to train each model separately and independently and determine the optimal hyperparameters of the model for each cluster. Learning through *k*-means clustering is referred to as learning with clustering in this study. Learning in Figure 2 without *k*-means clustering, i.e., learning the entire training data all together is referred to as learning without clustering.

### 3.2. Hybrid Method with Clustering

The proposed method first divides the training data into *k* groups through *k*-means clustering. Then for each data in the test data, an appropriate model is selected for the prediction as follows. Suppose that $\left\{C_{1},C_{2},\cdots ,C_{k}\right\}$ are the centroids of the groups in the partition of the training data. Given test data *X*, the distance between *X* and each of the centroids is measured as in (2),

(2) $$d\left(X,C_{i}\right)=∥X-C_{i}∥\;,\;i=1,2,\cdots ,k.$$

If the distance is minimized at $i=i^{*}$, that is

(3) $$d\left(X,C_{i^{*}}\right)=\min_{i}\left\{d\left(X,C_{i}\right)\;,\;i=1,2,\cdots ,k\right\},$$

then the learning model obtained from the $\left(i^{*}\right)$th cluster of the training data is applied for the prediction of *X*. The procedure is repeated for each of the test data as in Figure 4.

As shown in Section 4, different machine-learning methods exhibit different prediction results and no method is dominant in accuracy. Thus, a modification of ordinary machine-learning methods is considered, which is an ensemble method. When the obsolescence dates are predicted by three machine-learning methods, DT, RF, and GB, their average defines an obsolescence date (denoted by $y^{\mathrm{Hybrid}}$) by

(4) $$y^{\mathrm{Hybrid}}=\frac{1}{3}\left(y^{\mathrm{DT}}+y^{\mathrm{RF}}+y^{\mathrm{GB}}\right)\;,$$

where $y^{\mathrm{DT}}$, $y^{\mathrm{RF}}$, and $y^{\mathrm{GB}}$ are the obsolescence dates from DT, RF, and GB, respectively. The proposed hybrid method shows accurate and reliable results as presented in Section 4 and the application of the hybrid method is another novelty of this study.

Algorithm 1 summarizes the procedure of the proposed method. It should be noted that the algorithm is automatic so that no human intervention is required during the operation from the input data processing to the prediction of the obsolescence date.


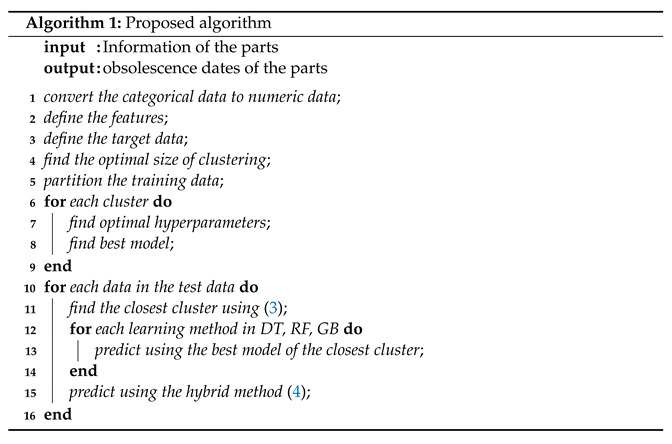


## 4. Data and Measures

We consider a case study to demonstrate the performance of the proposed machine-learning method in forecasting.

### 4.1. Data Collection and Problem Description

Because the prediction of the probability or the period of components obsolescence reduces the cost of purchase and maintenance, many defense industries and electronic component manufacturers have developed commercial component obsolescence prediction software. Many companies such as RAC, Z2Data, IHS, QTEC, Silicon Expert, Total Parts Plus, and AVCOM provide their own obsolescence prediction information by using various data and statistical methodologies, but the detailed methodologies or algorithms have not been disclosed. Particularly, in the case of software that provides the expected discontinuation period of parts, the error range is large or uncertain and it is provided without reference to any evidence. Therefore, it is difficult to use the obsolescence information from the commercial software as a basis for the study.

However, in the case of the parts that have already been discontinued, the part number can be obtained from QTEC along with the evidence that the discontinuation is certain, and the detailed characteristics and specifications of the part can be obtained from the Z2Data software. Among the parts available from those, the active discontinued parts in Zener diodes, varactors diodes, and bridge rectifier diodes with more than 10,000 cases have been selected and used in the study. In the case of passive components, the detailed characteristics and specifications of the parts are not diverse; thus they are excluded from this study. The data of Zener diodes, varactors diodes, and bridge rectifier diodes used in this study are provided by Leo Innovision Ltd.

The characteristics and specifications of the parts from various manufacturers are different in terms of the content and format. To standardize this, detailed technical specifications in the data sheets and test reports for each part have been thoroughly reviewed. Subsequently the characteristics common to most manufacturers that are considered important are selected as the features for each part. Through this process, 2366 Zener diodes, 350 varactors diodes, and 307 bridge rectifier diodes consisting of only discontinued parts among active electronic components while retaining different characteristics and specifications for each type are selected for the research. The diodes data from those three categories have 31, 44, and 41 features, respectively, and each dataset consists of numeric and categorical features. Table 1 lists the features for each category and the data type of the dataset used in this study.

Table 2 presents an example of Zener diode data. For simplicity, the values of only a few important features are shown. The data for the varactors or bridge rectifier diodes is similar and thus omitted.

**Figure 5:**
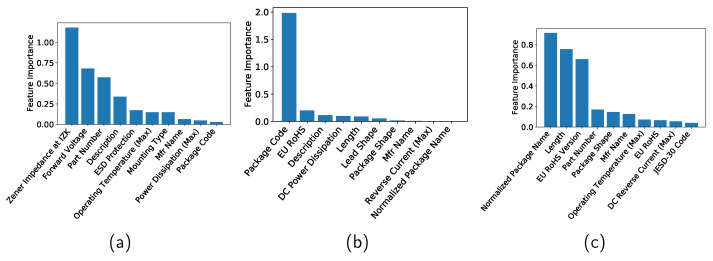
Feature importance of (**a**) Zener diodes, (**b**) varactors, and (**c**) bridge rectifier diodes when a decision tree is applied.

**Figure 6:**
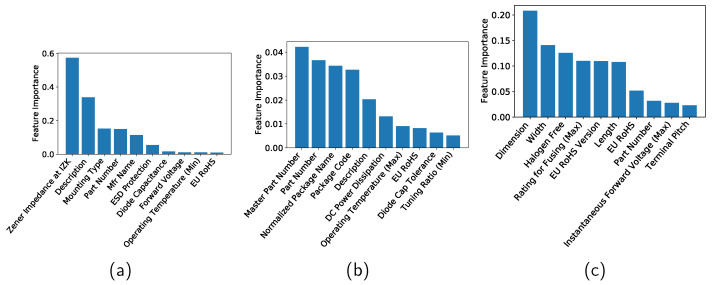
Feature importance of (**a**) Zener diodes, (**b**) varactors, and (**c**) bridge rectifier diodes when a random forest is applied.

**Figure 7:**
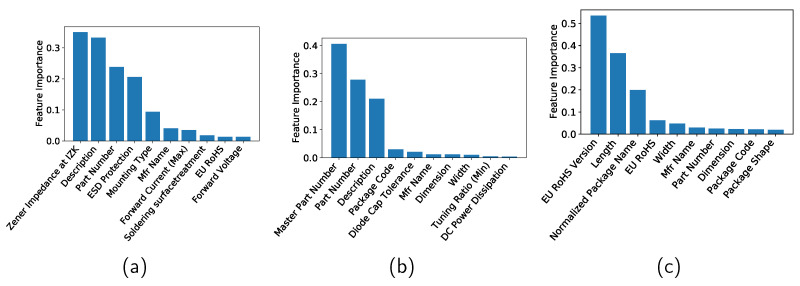
Feature importance of (**a**) Zener diodes, (**b**) varactors, and (**c**) bridge rectifier diodes when gradient boosting is applied.

The features in Table 1 have different contributions to machine learning. Feature importance refers to techniques that assign a score to input features based on how useful they are at predicting a target variable. Although there are many types and sources of feature importance scores, the feature importance is quantified in the current study by the permutation feature importance, which is a model inspection technique that can be used for any fitted estimator [24]. It is defined to be the decrease in a model score when a single feature value is randomly shuffled. This procedure breaks the relationship between the feature and the target; thus the drop in the model score is indicative of how much the model depends on the feature. See [24] for more information. In this study, the features are standardized by removing the mean and scaling to unit variance and then the feature importance is computed by using the $R^{2}$ score as the scoring parameter of the permutation importance function. Figure 5a–c show the top 10 features for the Zener diodes, varactors, and bridge rectifier diodes, respectively, when the DT method is applied. Figure 6 and Figure 7 show the importances when the RF and GB methods are applied, respectively.

Table 3 presents the statistics of the features of the Zener diodes. The count, mean and std represent the number, mean, and standard deviation of the data, respectively. Min and max are the minimum and maximum values, respectively and 25%, 50%, and 75% are quartiles, which divide the data points into four parts. The statistics for the varactors and bridge rectifier diodes are shown in Table 4 and Table 5, respectively.

### 4.2. Hyperparameters

**Figure 8:**
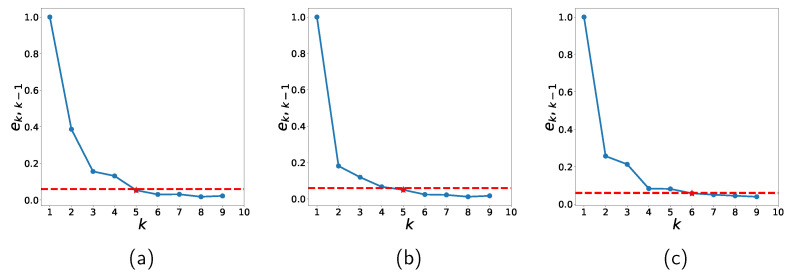
Accuracies with respect to different *k* values for (**a**) Zener diodes, (**b**) varactors, and (**c**) bridge rectifier diodes.

For the *k*-means to be effective, an appropriate number *k* of clusters should be estimated. As a preprocessing step, for each of k=1,2,⋯, training data is partitioned into *k* clusters and DT is applied to estimate the accuracy. *k* is increased until the improvement $|e_{k}-e_{k-1}|$ is small enough, where $e_{k}$ represents the MRE error defined in Section 5 with *k* clusters. That is, *k* is chosen such that

(5) $$e_{k,k-1}\equiv \alpha \left(e_{k}-e_{k-1}\right)<h,$$

where *h* is a threshold. $\alpha \equiv {\left(e_{2}-e_{1}\right)}^{-1}$ is introduced to avoid dependency on the dataset. Figure 8 shows $e_{k,k-1}$ in (5) for several *k* values. h=0.06 is used in this study and the optimal *k* for the datasets are listed in Table 6.

Each machine-learning method has hyperparameters and the hyperparameters used in this study are summarized in Table 7. The leftmost column in Table 7 represents the names of the parameters, which are taken from the scikit library [24]. For instance, DT in the current study considers 4 hyperparameters, i.e., min_samples_split, max_depth, max_sample_leaf, max_leaf_nodes. The column in the middle describes the definition of each hyperparameter and the values of the hyperparameter considered in this study are shown in the rightmost column. For instance, the maximum depth of the tree (max_depth) for DT is one of 2, 4, 6, and 8. Then, one creates a grid of all possible hyperparameter combinations. For instance, in case of DT, all combinations from 5 values of min_samples_split, 4 values of max_depth, 9 values of min_samples_leaf, and 4 values of max_leaf_nodes are created, and DT is trained with each one of them to find the best parameters. Model tuning with such a grid search is performed for other models similarly with the values in Table 7.

## 5. Results and Discussion

To compare the performance of different methods, the accuracy is measured by the mean relative error (MRE):

(6) $$\mathrm{MRE}=\frac{1}{N}\sum_{i=1}^{N}\left|\frac{y_{i}-{\tilde{y}}_{i}}{y_{i}}\right|,$$

and the root mean squared relative error (RMSRE):

(7) $$\mathrm{RMSRE}=\sqrt{\frac{1}{N}\sum_{i=1}^{N}{\left(\frac{y_{i}-{\tilde{y}}_{i}}{y_{i}}\right)}^{2}},$$

where $y_{i}$ is the actual value and ${\tilde{y}}_{i}$ is the predicted value. *N* is the number of predictions.

If a machine-learning method is not applied, statistical methods can be applied for the prediction of the obsolescence date. For the expected value of the obsolescence date, the sample mean of the observed, i.e., known obsolescence dates from the training data can be used as a prediction value, which will be referred to as “Statistic” below. That is, Statistic is defined by (8)

(8) $$\mathrm{Statistic}=\frac{1}{N_{\mathrm{tr}}}\sum_{i=1}^{N_{\mathrm{tr}}}y_{i},$$

where $N_{\mathrm{tr}}$ is the number of the training data, which can be used as a naive prediction value for the test data.

**Figure 9:**
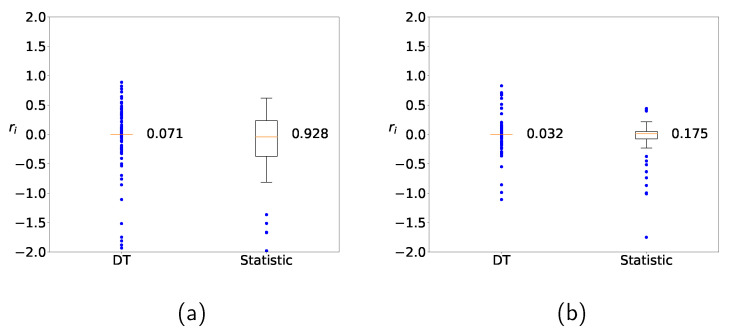
Distribution of predicted values for Zener diodes with the DT method (**a**) without clustering and (**b**) with clustering.

We first determine whether learning with clustering produces any improvement over learning without clustering. Figure 9 shows the distribution of the relative error of the prediction

(9) $$r_{i}\equiv \frac{y_{i}-{\tilde{y}}_{i}}{y_{i}}$$

for the Zener diode data when DT and the naive statistic are applied. Figure 9a shows the distribution without clustering and Figure 9b with clustering, respectively. The deviations from DT are smaller and the corresponding predictions are closer to the actual values than the naive approach. It should be noted that the predicted values from DT with clustering are closer to the actual values than those without clustering. Clustering is observed to reduce the variation and improve the prediction accuracy.

**Figure 10:**
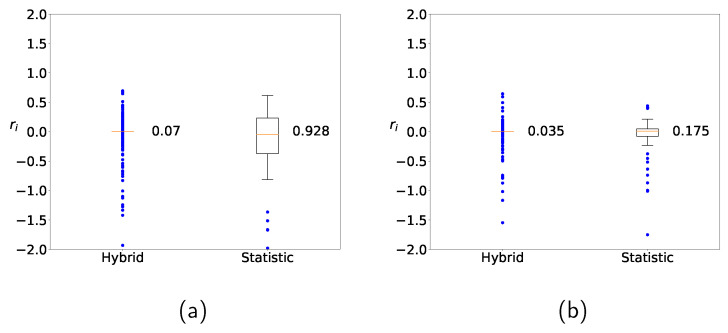
Distribution of predicted values for Zener diodes with the hybrid method (**a**) without clustering and (**b**) with clustering.

Figure 10 shows the distributions of $r_{i}$ in (9) by using the hybrid method (a) without clustering and (b) with clustering. Similarly to Figure 9, the range of the distributed values from the hybrid method is narrower than that from the naive statistic, and the result from the hybrid method with clustering is superior to the result from the hybrid method without clustering. Similar trends are observed for other machine-learning methods or other datasets as well (not shown), and it is empirically supported that clustering leads to improvement.

**Figure 11:**
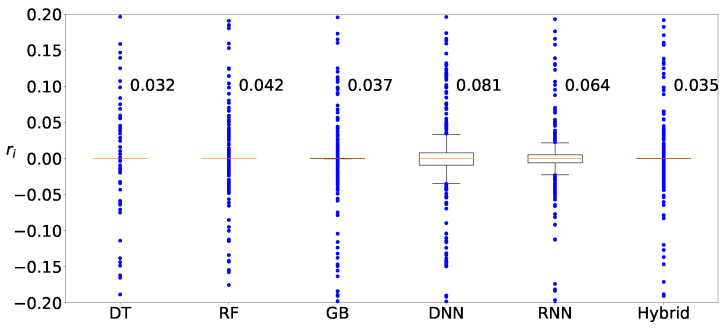
Distribution of predicted values for Zener diodes by using DT, RF, GB, DNN, RNN, and hybrid methods with clustering.

**Figure 12:**
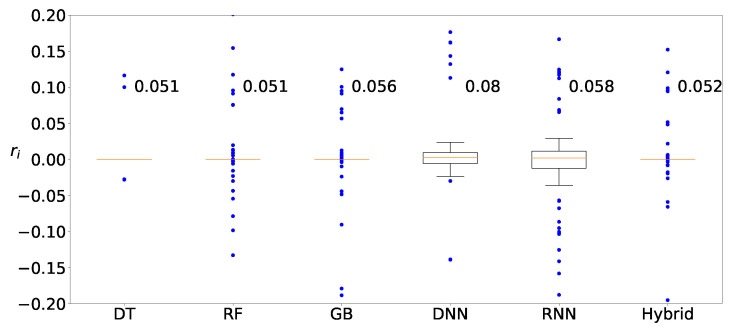
Distribution of predicted values for varactors by using DT, RF, GB, DNN, RNN, and hybrid methods with clustering.

**Figure 13:**
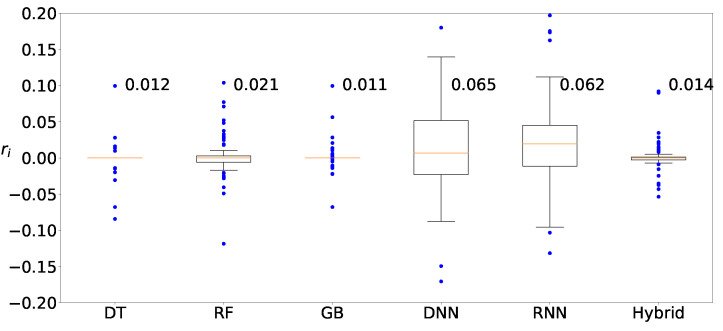
Distribution of predicted values for bridge rectifier diodes by using DT, RF, GB, DNN, RNN, and hybrid methods with clustering.

Next, we determine the machine-learning method that produces the best prediction result. Figure 11 shows the distributions of the deviation of the prediction from various machine-learning methods, DT, RF, GB, DNN, RNN, and hybrid, when clustering is applied to Zener diodes. Four machine-learning methods, DT, RF, GB, and hybrid, result in similar prediction distributions, whereas the results from two deep-learning methods, DNN and RNN, are slightly worse than those from the machine-learning methods. Figure 12 and Figure 13 show the results of the varactors and bridge rectifier diodes, and similar trends are observed. One of the reasons for the poor results from the deep-learning methods may be result from insufficient data. In fact, deep learning is superior to ordinary shallow machine learning if the number of data is large enough. However, the data for the current case study are insufficient and the ordinary shallow machine-learning produces better results than the deep learning in this study.

Subsequently, we compare the prediction accuracy with respect to two measures, MRE and RMSRE. Table 8 presents the MRE errors of the training data with and without clustering. It shows that the errors from the naive statistic prediction and two deep-learning methods, the DNN and RNN methods are larger than those of the other shallow machine-learning methods and that training with GB overfits the given training data.

Table 9 lists the MRE error of the test data with and without clustering. The predictions from all the machine-learning or deep-learning methods with or without clustering are better than the naive statistic prediction and the four shallow machine-learning methods, DT, RF, GB, and hybrid methods produce better results than DNN and RNN for for all the three categories. Deep learning methods produce good regression accuracies in many applications, but they have difficulty in finding right parameters in this study owing to the lack of data.

**Figure 14:**
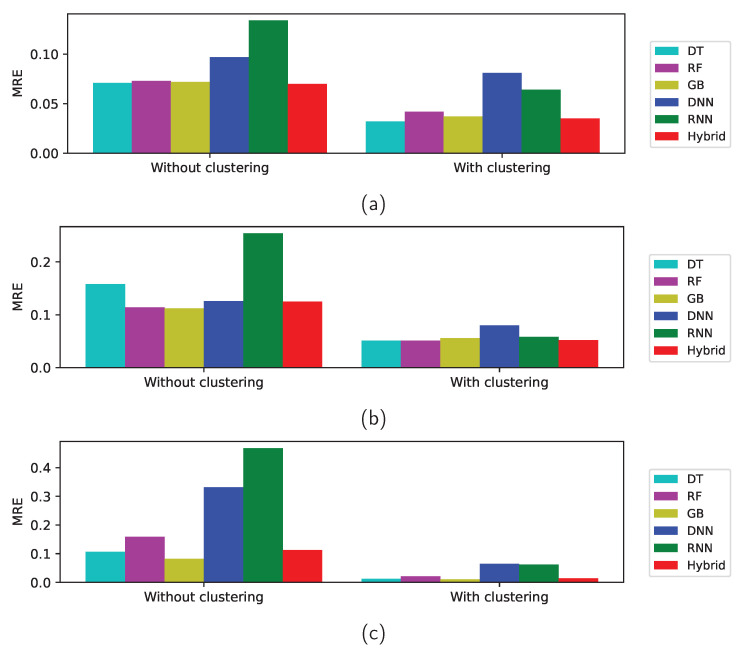
MRE of the test data with and without clustering for (**a**) Zener diodes, (**b**) varactors, and (**c**) bridge rectifier diodes.

Although the prediction of Statistic from clustering is improved over the prediction without clustering, the results from the machine learning still dominate. When clustering is applied, the errors from the four shallow learning methods are smaller than those from deep-learning methods. Among shallow machine-learning methods, the DT, GB, and hybrid methods give good predictions for the Zener diodes and bridge rectifier diodes, whereas the DT, RF, and hybrid methods give good predictions for the varactors. Because the data in each cluster from the *k*-means algorithm has less variation than the entire data, the machine-learning model trained with the clusters represents the data better than a single model trained with the entire data and thus the accuracies of the models with clustering are better than those without clustering even when the same model is applied. It should be noted that the hybrid method produces good accuracy regardless of the category or the training method, which implies that the hybrid method is reliable. Figure 14a presents the MRE of the test data with and without clustering for Zener diodes, which shows that model training with unsupervised clustering algorithm improves the prediction accuracy and reduces the errors. Similar reduction in MRE is observed in the varactors as in Figure 14b and bridge rectifier diodes as in Figure 14c.

Table 10 lists the RMSRE errors of the training data with and without clustering. Similarly to Table 8, the errors from the naive statistic, DNN, and RNN methods are larger than the others and training with GB seems to overfit.

**Figure 15:**
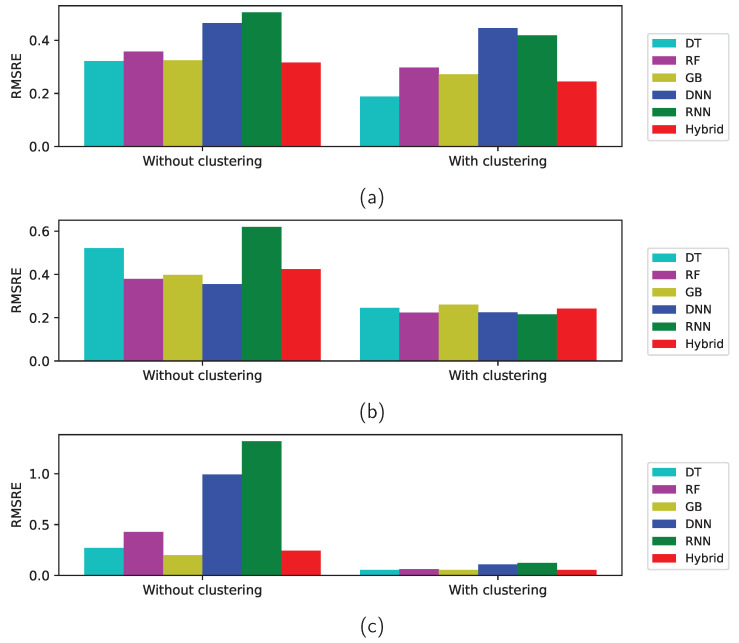
RMSRE of the test data with and without clustering for (**a**) Zener diodes, (**b**) varactors, and (**c**) bridge rectifier diodes.

Table 11 lists the RMSRE errors of the test data with and without clustering. The predictions from all the machine-learning methods without clustering are better than the naive statistic prediction for the Zener diodes and varactors. In case of the bridge rectifier diodes, the Statistic and RNN methods without clustering result in large errors. In fact, the RMSRE errors from RNN method are large for all the three categories. The RMSRE errors from the models with clustering are smaller than those without clustering as in Table 12. The RMSRE errors from the deep-learning methods, DNN and RNN, with clustering are as small as those from the other methods for the varactors. Although the trends of the results from the RMSRE are quite similar to those from the MRE, the errors from the RMSRE are relatively larger than those from the MRE because some errors are large owing to an insufficient amount of data and the RMSRE is dependent more on such values than the MRE. Figure 15 presents the RMSRE of the test data with and without clustering for the Zener diodes, varactors, and bridge rectifier diodes, respectively. The figure shows again that unsupervised clustering algorithm improves the prediction accuracy of the supervised regression models as observed in Figure 14.

**Figure 16:**
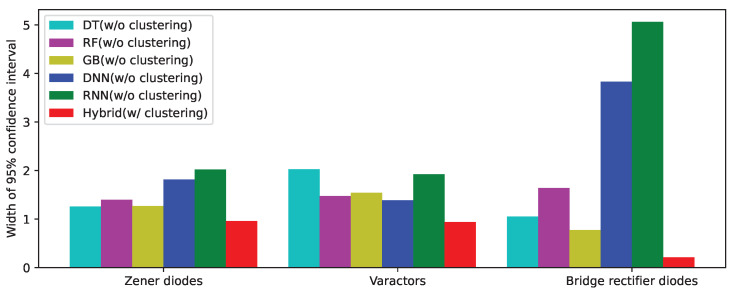
The widths of the 95% confidence intervals of the predicted values using various methods for the Zener diodes, varactors, and bridge rectifier diodes.

Table 13 lists the widths of the 95% confidence intervals of the predicted values using various methods. As shown in the Table 13, the size of the confidence interval of the hybrid method with clustering is much smaller than that of the method without clustering. Therefore, it can be inferred that the estimate using the proposed method with clustering is more stable and accurate. As an example, for the bridge rectifier diodes data, the width of the confidence interval of the predicted value using an RNN is 24 times wider, and in the case of using an RF, the width is 7.8 times wider than that obtained by using the proposed hybrid method. Figure 16 presents the widths of the 95% confidence interval using a bar graph, which shows the variation of the prediction accuracy of various machine-learning methods. The bar corresponding to the proposed hybrid method with clustering (red) is shorter than the others for all the three categories, which confirms the superiority of the proposed method.

## 6. Conclusions

This paper proposed an accurate and reliable method for the prediction of the obsolescence date of the components of the diodes based on the *k*-means method and a hybrid ensemble method. It is the novelty of the study to apply the unsupervised clustering method to the supervised regression problem to improve the prediction. The *k*-means unsupervised clustering algorithm partitioned the entire set into clusters of similar data. The proposed method trained with similar data in each cluster demonstrated better predictions than the single model trained with the entire set regardless of the category of the diodes even when a sufficient amount of data was not provided whereby ordinary shallow or deep-learning methods would face difficulties in realizing accurate forecasts. The hybrid method including several regression techniques made further improvements in prediction accuracy.

There are two research directions from the current proposed model. One is the combination of unsupervised clustering and deep-learning models with many hidden layers and sufficiently many data samples, which was not supported in the current study. It is expected that the accuracy of the deep-learning method will be improved when training is performed with similar data samples. The other direction is to improve the clustering method. Although the *k*-means algorithm is a good clustering method, there still exist areas for continued development such as sensitivity to initial values or hyperparameter tuning. Moreover, because unsupervised clustering method partitions the entire data into disjointed clusters, some samples near a boundary are assigned to clusters, which are not intuitively appropriate. If there can be a way to handle those data properly and assign them to appropriate clusters, the prediction will be improved even further.

The proposed method is applied to the obsolescence of electric diodes in this study, which can be applied to various fields from the obsolescence of other components to any regression problems in sciences such as financial market prediction.

## Figures and Tables

**Table 1:** Features of the diode data from three categories.

Category	Type	Features
Zener diodes	Numeric	Power Dissipation, Reverse Zener Voltage (Min), Reverse Zener Voltage (Max), Test Current, Zener Impedance (Max), Zener Impedance at IZK, Maximum Zener Current, Reverse Leakage Current at VR, Reverse Voltage, Forward Voltage, Voltage Tolerance, Forward Current, Diode Capacitance, Operating Temperature (Min), Operating Temperature (Max), Number of Terminals
	Categorical	Part Number, Mfr Name, Description, Polarity, ESD Protection, Temperature Coefficient, EU RoHS, Halogen Free, Package Code, Soldering surface treatment, Mounting Type, JESD-30 Code, Package Body Material, Package Shape, Package Style, Terminal Form, Terminal Position, Temperature Grade, Part Status, Part Introduction, Obsolete Date(LTB Date)
Varactors	Numeric	Number of Terminals, Technology, Breakdown Voltage, Forward Current (Max), Reverse Current (Max), Capacitance (Min), Capacitance (Max), Capacitance (Nom), Diode Cap Tolerance, Operating Temperature (Min), Operating Temperature (Max), DC Power Dissipation, Quality Factor, Tuning Ratio, V-HBM, V-CDM, V-MM, Halogen Free, Number of Terminals, Length, Width, Terminal Pitch, Package Equivalence Code, DLA Qualification, Qualifications, Screening Level/Reference Standard
	Categorical	Master Part Number, Part Number, Mfr Name, Description, Configuration, EU RoHS, EU RoHS Version, China RoHS, REACH Compliant, Package Code, Dimension, Normalized Package Name, Mounting Type, Lead Shape, JESD-30 Code, Package Body Material, Package Shape, Package Style, Terminal Form, Terminal Position, Surface Mount, Temperature Grade, Part Status
Bridge rectifier diodes	Numeric	Number of Phases, Number of Terminals, Repetitive Peak Reverse Voltage, Root Mean Squared Voltage, DC Blocking Voltage, Instantaneous Forward Voltage, Peak Forward Surge Current, Average Rectified Output Current, DC Reverse Current, I2T Rating for Fusing, Operating Temperature (Min), Operating Temperature (Max), V-HBM, V-CDM, V-MM, Number of Terminals, Length, Width, Terminal Pitch, Package Equivalence Code, Qualifications, Screening Level/Reference Standard
	Categorical	Master Part Number, Part Number, Mfr Name, Description, EU RoHS, EU RoHS Version, China RoHS, Halogen Free, REACH Compliant, Package Code, Dimension, Normalized Package Name, Mounting Type, Lead Shape, JESD-30 Code, Package Body Material, Package Shape, Package Style, Terminal Form, Terminal Position, Surface Mount, DLA Qualification, Temperature Grade, Part Status

**Table 2:** Example of data collected for a Zener diode.

Feature	Value
Zener Impedance at IZK	2000.0
Forward Voltage	1.2
Part Number	1N4761A
Description	ZENER DIODE, 1W, 75V@3MA, 5%
ESD Protection	Unknown
Operating Temperature (Max)	200
Mounting Type	Through Hole
Mfr Name	CENTRAL SEMICONDUCTOR CORP.
Power Dissipation (Max)	1.0
Package Code	DO-41

**Table 3:** Statistics for the features of Zener diodes.

	Count	Mean	Std	Min	25%	50%	75%	Max
Power Dissipation (Max)	2366	2.52	2.49	0.12	0.50	1.0	5.00	10.0
Reverse Zener Voltage (Min)	1051	26.70	36.66	1.80	5.60	12.4	28.50	190.0
Reverse Zener Voltage (Max)	2366	42.23	52.44	1.80	8.53	20.0	53.16	270.0
Test Current	2366	32.61	61.26	0.25	5.00	10.0	30.00	640.0
Zener Impedance (Max)	2366	110.81	240.05	1.00	9.00	30.0	100.00	2500.0
Zener Impedance at IZK	1936	1129.16	1361.70	60.00	400.00	700.0	1300.00	8000.0
Maximum Zener Current	1244	200.44	288.66	1.54	31.60	85.0	264.00	2380.0
Reverse Leakage Current at VR	2366	8.73	22.78	0.05	1.00	2.0	5.00	300.0
Reverse Voltage	2366	31.16	39.76	0.50	6.00	15.0	38.80	206.0
Forward Voltage	1811	1.29	0.21	0.90	1.20	1.2	1.50	1.5
Voltage Tolerance (Max)	2366	4.62	2.56	1.00	2.38	5.0	5.00	20.0
Forward Current (Max)	1811	437.88	395.93	2.00	200.00	200.0	1000.00	1000.0
Diode Capacitance	157	178.23	148.24	19.00	70.00	130.0	225.00	450.0
Operating Temperature (Min)	2366	−62.52	4.32	−65.00	−65.00	−65.0	−65.00	−55.0
Operating Temperature (Max)	2366	171.56	16.36	125.00	150.00	175.0	175.00	200.0
Number of Terminals	2366	1.98	0.49	0.00	2.00	2.0	2.00	4.0

**Table 4:** Statistics for the features of varactors.

	Count	Mean	Std	Min	25%	50%	75%	Max
Number of Terminals	350	2.21	0.41	2.00	2.00	2.00	2.00	3.00
Breakdown Voltage (Max)	350	32.76	13.91	6.00	25.00	30.00	32.00	65.00
Forward Current (Max)	200	142.25	86.01	10.00	20.00	200.00	200.00	250.00
Reverse Current (Max)	350	0.32	3.77	0.00	0.02	0.02	0.02	50.00
Capacitance (Min)	308	21.61	21.06	0.70	5.94	14.40	29.78	98.00
Capacitance (Max)	308	27.15	26.68	0.88	7.40	18.18	36.30	120.00
Capacitance (Nom)	350	25.33	24.06	0.80	6.80	18.00	33.00	100.00
Diode Cap Tolerance	237	10.95	6.33	2.00	5.00	10.00	20.00	30.23
Operating Temperature (Min)	306	−60.20	5.00	−65.00	−65.00	−65.00	−55.00	−55.00
Operating Temperature (Max)	347	152.84	18.73	85.00	150.00	150.00	175.00	175.00
DC Power Dissipation	282	332.64	67.22	200.00	250.00	330.00	400.00	400.00
Quality Factor (Min)	291	393.20	470.42	75.00	200.00	300.00	450.00	2900.00
Tuning Ratio (Min)	329	4.70	4.03	1.50	2.80	3.20	5.00	35.00
Number of Terminals	350	2.21	0.41	2.00	2.00	2.00	2.00	3.00
Length	299	2.15	0.77	1.00	1.70	2.42	2.42	4.83
Width	299	1.91	0.77	0.60	1.30	2.42	2.42	3.68
Terminal Pitch	66	1.11	0.62	0.65	0.92	0.92	0.92	2.54

**Table 5:** Statistics for the features of bridge rectifier diodes.

	Count	Mean	Std	Min	25%	50%	75%	Max
Number of Phases	307	1.00	0.00	1.00	1.00	1.00	1.0	1.0
Number of Terminals	307	3.99	0.11	2.00	4.00	4.00	4.0	4.0
Repetitive Peak Reverse Voltage (Max)	307	507.23	317.89	30.00	200.00	600.00	800.0	1000.0
Root Mean Squared Voltage (Max)	290	349.01	227.75	35.00	140.00	330.00	560.0	700.0
DC Blocking Voltage (Max)	307	507.23	317.89	30.00	200.00	600.00	800.0	1000.0
Instantaneous Forward Voltage (Max)	307	1.07	0.12	0.42	1.00	1.10	1.1	2.7
Peak Forward Surge Current (Max)	307	149.04	132.59	30.00	50.00	60.00	300.0	400.0
Average Rectified Output Current (Max)	307	9.92	13.69	0.50	1.50	2.00	15.0	50.0
DC Reverse Current (Max)	307	10.10	56.88	5.00	5.00	5.00	10.0	1000.0
Rating for Fusing (Max)	215	167.32	240.06	3.00	10.00	15.00	373.0	664.0
Operating Temperature (Min)	291	−55.22	4.65	−65.00	−55.00	−55.00	−55.0	−40.0
Operating Temperature (Max)	307	148.27	9.80	125.00	150.00	150.00	150.0	175.0
Number of Terminals	303	3.99	0.11	2.00	4.00	4.00	4.0	4.0
Length	307	15.93	8.12	3.00	8.85	14.78	23.2	30.0
Width	307	10.72	8.88	3.40	4.60	6.40	15.2	29.0
Terminal Pitch	307	7.60	5.23	2.50	3.86	5.10	10.8	18.1

**Table 6:** Optimal *k* values for the three categories.

Category	*k*
Zener diodes	5
Varactors	5
Bridge rectifier diodes	6

**Table 7:** Hyperparameters for the machine learning methods used in this study.

**DT**	**Definition**	**Values**
min_samples_split	The minimum number of samples required to split an internal node	None, 2, 4, 6, 8
max_depth	The maximum depth of the tree	2, 4, 6, 8
min_samples_leaf	The minimum number of samples required to be at a leaf node	2, 3, 4, …, 10
max_leaf_nodes	The maximum number of leaf nodes	None, 20, 40, 60
**RF**	**Definition**	**Values**
min_samples_split	The minimum number of samples required to split an internal node	2, 3, 4, 5
n_estimators	The number of trees in the forest	100, 150, 200
max_features	The number of features to consider when looking for the best split	auto, sqrt, log2
**GB**	**Definition**	**Values**
learning_rate	Learning rate	0.01, 0.1, 0.2
subsample	The fraction of samples to be used for fitting the individual base learners	0.5, 0.6, 0.7, 0.8, 0.9, 1
n_estimators	The number of boosting stages to perform	100, 200, 300, 400, 500
max_depth	The maximum depth of the individual regression estimators	2, 4, 6, 8, 10
**DNN**	**Definition**	**Values**
unit	The dimensionality of the output space	32, 64
optimizer	The optimizer which adjusts model weights to minimize the loss function	Adam, Nadam, RMSprop
dropout	The fraction of the units to drop for the linear transformation of the inputs	0, 0.1, 0.01
**RNN**	**Definition**	**Values**
unit	The dimensionality of the output space	32, 64
optimizer	The optimizer which adjusts model weights to minimize the loss function	Adam, Nadam, RMSprop
dropout	The fraction of the units to drop for the linear transformation of the inputs	0, 0.1, 0.01

**Table 8:** MRE of the training data with and without clustering.

	Method	Zener Diodes	Varactors	Bridge Rectifier Diodes
	Statistic	0.730	0.911	0.581
	DT	0.000	0.040	0.020
	RF	0.024	0.036	0.068
Without Clustering	GB	0.000	0.000	0.000
	DNN	0.065	0.101	0.409
	RNN	0.095	0.198	0.469
	Hybrid	0.008	0.023	0.029
	Statistic	0.130	0.084	0.071
	DT	0.006	0.000	0.001
	RF	0.012	0.018	0.011
With Clustering	GB	0.001	0.001	0.000
	DNN	0.040	0.054	0.041
	RNN	0.037	0.046	0.046
	Hybrid	0.006	0.006	0.004

**Table 9:** MRE of the test data with and without clustering.

	Method	Zener Diodes	Varactors	Bridge Rectifier Diodes
	Statistic	0.928	0.933	0.513
	DT	0.071	0.158	0.107
	RF	0.073	0.114	0.159
Without Clustering	GB	0.072	0.112	0.082
	DNN	0.097	0.126	0.332
	RNN	0.134	0.254	0.468
	Hybrid	0.070	0.125	0.113
	Statistic	0.175	0.087	0.068
	DT	0.032	0.051	0.012
	RF	0.042	0.051	0.021
With Clustering	GB	0.037	0.056	0.011
	DNN	0.081	0.080	0.065
	RNN	0.064	0.058	0.062
	Hybrid	0.035	0.052	0.014

**Table 10:** RMSRE of the training data with and without clustering.

	Method	Zener Diodes	Varactors	Bridge Rectifier Diodes
	Statistic	1.913	1.711	1.330
	DT	0.000	0.213	0.102
	RF	0.109	0.120	0.165
Without Clustering	GB	0.000	0.000	0.000
	DNN	0.302	0.323	1.148
	RNN	0.302	0.448	1.394
	Hybrid	0.036	0.097	0.069
	Statistic	0.500	0.238	0.102
	DT	0.058	0.002	0.008
	RF	0.075	0.102	0.023
With Clustering	GB	0.004	0.003	0.000
	DNN	0.211	0.203	0.066
	RNN	0.216	0.227	0.078
	Hybrid	0.038	0.035	0.008

**Table 11:** RMSRE of the test data with and without clustering.

	Method	Zener Diodes	Varactors	Bridge Rectifier Diodes
	Statistic	2.751	1.668	1.230
	DT	0.322	0.522	0.270
	RF	0.358	0.380	0.427
Without Clustering	GB	0.325	0.398	0.198
	DNN	0.465	0.355	0.993
	RNN	0.505	0.620	1.318
	Hybrid	0.316	0.425	0.243
	Statistic	0.778	0.221	0.098
	DT	0.188	0.246	0.054
	RF	0.297	0.223	0.062
With Clustering	GB	0.272	0.261	0.053
	DNN	0.446	0.225	0.109
	RNN	0.419	0.215	0.124
	Hybrid	0.245	0.242	0.055

**Table 12:** MRE of the test data with and without clustering.

	Method	Zener Diodes	Varactors	Bridge Rectifier diodes
	Statistic	0.928	0.933	0.513
	DT	0.071	0.158	0.107
	RF	0.073	0.114	0.159
Without Clustering	GB	0.072	0.112	0.082
	DNN	0.097	0.126	0.332
	RNN	0.134	0.254	0.468
	Hybrid	0.070	0.125	0.113
	Statistic	0.175	0.087	0.068
	DT	0.032	0.051	0.012
	RF	0.042	0.051	0.021
With Clustering	GB	0.037	0.056	0.011
	DNN	0.081	0.080	0.065
	RNN	0.064	0.058	0.062
	Hybrid	0.035	0.052	0.014

**Table 13:** Comparison of the widths of the 95% confidence intervals of the predicted values using various methods.

		Zener Diodes	Varactors	Bridge Rectifier Diodes
	DT	1.259921	2.027222	1.052013
	RF	1.396787	1.472401	1.641815
Without Clustering	GB	1.269164	1.544143	0.773789
	DNN	1.814925	1.384894	3.830107
	RNN	2.020643	1.920797	5.063853
With Clustering	Hybrid	0.956952	0.940740	0.213291

## Data Availability

Data sharing not applicable.

## Abbreviations

DT	Decision tree
RF	Random forest
GB	Gradient boosting
DNN	Deep neural network
RNN	Recurrent neural network
MRE	Mean relative error
RMSRE	Root mean squared relative error
